# Quality of Life of Elderly Residents of Old-Age Homes: A Cross-Sectional Study From Tripura

**DOI:** 10.7759/cureus.112554

**Published:** 2026-07-12

**Authors:** Bhawna C Datta, Pramod K Yadav, Bibhash Datta

**Affiliations:** 1 Occupational Therapy, Jaipur Occupational Therapy College, Maharaj Vinayak Global University, Jaipur, IND; 2 Community Medicine, Tripura Medical College and Dr. B.R. Ambedkar Memorial Teaching Hospital, Agartala, IND

**Keywords:** care of the elderly, geriatric, geriatric mental health, old age home, quality of life

## Abstract

Background and aim

Quality of life (QOL) among older adults is an important indicator of the health and well-being of this vulnerable population. This study aimed to assess the QOL of older adults residing in old-age homes.

Methods

This facility-based cross-sectional study was conducted between January and February 2024 among 79 elderly residents aged ≥60 years from five randomly selected old-age homes in Agartala. Data were collected using a semi-structured questionnaire and the World Health Organization Quality of Life-BREF (WHOQOL-BREF) instrument. Domain scores were calculated according to WHO guidelines. Independent-samples *t* test, one-way ANOVA, and multiple linear regression were used to compare QOL scores and identify independent predictors of QOL. A p-value <0.05 was considered statistically significant.

Results

The social relationships domain recorded the highest mean QOL score (67.3 ± 11.0), whereas the psychological domain had the lowest score (44.2 ± 11.7). Participants aged ≤70 years had significantly higher physical domain scores than those aged >70 years (p = 0.020). Higher socioeconomic status was independently associated with better psychological QOL (B = 11.63, 95% CI: 2.78-20.48; p = 0.011), whereas participants who lived in joint families had significantly higher social relationships domain scores (B = 8.79, 95% CI: 0.54-17.03; p = 0.037). No significant associations were observed with gender, religion, marital status, comorbidity status, or type of old-age home.

Conclusions

Older adults residing in old-age homes in Agartala exhibited a relatively high QOL in the social relationships domain, although their psychological well-being was comparatively compromised. These findings provide region-specific evidence that could guide the development of comprehensive geriatric care and supportive services for older adults living in old-age homes.

## Introduction

Aging is an unavoidable physiological process that encompasses physical, social, and psychological aspects. As people age, they experience physiological and physical changes that can limit or hinder their ability to engage in certain activities. These changes often lead to feelings of unhappiness and have a significant impact on their quality of life (QOL), particularly with respect to their health [1,2]. During the later stages of life, various adverse factors, such as low socioeconomic status, limited education, and gender, along with functional impairments, reduced daily activity levels, diminished mobility, fear of falling due to mobility and vision problems, sleep disturbances, cognitive changes, and other disabling conditions, contribute to a decline in both QOL and social engagement [3].

QOL is a multidimensional concept encompassing physical health, psychological well-being, social relationships, and environmental conditions. Assessment of QOL provides a comprehensive understanding of healthy aging beyond the mere absence of disease and serves as an important indicator for planning geriatric health and social care services. Among institutionalized older adults, QOL is influenced not only by individual health status but also by the living environment, opportunities for social participation, access to healthcare, and institutional support systems [4-7].

Several studies from other countries and different regions of India have evaluated the QOL of older adults residing in community or institutional settings [2,6-15]. However, these findings may not be directly applicable to northeastern India because of substantial regional differences in socioeconomic conditions, family structure, cultural practices, healthcare accessibility, and the availability of geriatric care services. Although a few studies have examined QOL among community-dwelling older adults in Tripura, evidence regarding elderly residents of old-age homes is scarce. Given the increasing reliance on institutional care for older adults and the limited region-specific evidence from Tripura, understanding the QOL of this vulnerable population is important for developing contextually appropriate interventions and informing local health and social welfare policies.

Therefore, the present study aimed to assess the QOL of elderly residents of old-age homes in Agartala, Tripura, and to identify sociodemographic factors associated with different domains of QOL.

## Materials and methods

Study design, setting, duration, and population

This facility-based observational cross-sectional study was conducted between January and February 2024 among adults aged ≥60 years residing in registered old-age homes in Agartala, Tripura, India.

Inclusion and exclusion criteria

Individuals aged ≥60 years residing in the selected old-age homes were eligible for inclusion in the study, except those who were severely ill or declined to provide informed consent.

Sample size

The sample size was calculated using the formula for estimating a population mean:

$n=\frac{Z^{2}\sigma^{2}}{d^{2}}$

To achieve a 99% confidence level, a Z value of 2.58 was applied. The SD (σ) of the social relationships domain score among older adults was taken as 15.3, based on findings from a previous study [13]. An absolute precision (d) of 5% was considered. The minimum required sample size was calculated as 62.3, which was increased by 20% to account for potential nonresponse or incomplete data, resulting in a minimum required sample size of 75 participants.

Sampling method

A two-stage sampling approach was used. In the first stage, a list of all eight registered old-age homes in Agartala was obtained from the Directorate of Social Welfare and Social Education, Government of Tripura. The sampling frame comprised three government-run and five nongovernmental organization (NGO)-managed old-age homes. Using simple random sampling, five old-age homes (two government-run and three NGO-managed) were selected for the study. In the second stage, all residents aged ≥60 years in the selected old-age homes were screened for eligibility. Eligible residents who met the inclusion criteria and provided written informed consent were consecutively recruited. During the study period, 79 eligible residents consented to participate and were included in the final analysis.

Data collection

Data were collected using a semi-structured questionnaire (Appendix A). The questionnaire consisted of two sections. Section I captured participants’ sociodemographic and health-related information. No personally identifiable information was collected from the participants. Section II assessed QOL using the World Health Organization Quality of Life-BREF (WHOQOL-BREF) instrument, a validated questionnaire comprising 26 items across four domains: physical health, psychological health, social relationships, and environment [4]. The socioeconomic status of the participants was assessed using the Modified B.G. Prasad Socioeconomic Classification (2023 revision) [16].

Statistical analysis

All collected data were managed using Microsoft Excel (Microsoft Corporation, Redmond, WA, USA) and analyzed using IBM SPSS Statistics for Windows, version 26.0 (released 2018; IBM Corp., Armonk, NY, USA). Proportions and percentages were calculated for qualitative variables, and means with SDs were calculated for quantitative variables. WHOQOL-BREF scores were calculated according to the WHO scoring guidelines, with raw domain scores transformed to a 0-100 scale, where higher scores indicate better QOL [4]. The mean WHOQOL-BREF domain scores and the two general WHOQOL items (overall QOL and overall health satisfaction) were compared across sociodemographic characteristics using independent-samples t tests or one-way ANOVA, as appropriate.

To identify independent sociodemographic predictors of QOL, separate multiple linear regression models were constructed for each WHOQOL-BREF domain and the two general WHOQOL items (overall QOL and overall health satisfaction). Variables with p < 0.20 in the univariate analysis, together with variables considered clinically or epidemiologically relevant based on the previous literature, were included in the multivariable regression models. Before model fitting, the assumptions of multiple linear regression, including linearity, normality of residuals, homoscedasticity, independence of errors, and absence of multicollinearity, were assessed using diagnostic plots and statistical measures and were found to be satisfactory. Statistical significance was set at p < 0.05.

Ethical considerations

The study was initiated after approval was obtained from both the Scientific Review Committee and the University Ethics Committee of Maharaj Vinayak Global University. Written informed consent was obtained from all participants before data collection.

## Results

Sociodemographic characteristics

A total of 79 participants were included, with a mean age of 75.0 ± 9.3 years (range, 60-90 years). The majority were aged 71-80 years (35.4%, 28/79), female (68.4%, 54/79), Hindu (75.9%, 60/79), widowed (65.8%, 52/79), and from the lower middle socioeconomic class (34.2%, 27/79). All participants had at least one comorbidity; among them, 41 (51.9%) had a single comorbidity and 38 (48.1%) had multiple comorbidities. Nearly three-fifths (59.5%, 47/79) of the participants resided in NGO-managed old-age homes, whereas 40.5% (32/79) resided in government-run facilities. The detailed sociodemographic characteristics of the participants are presented in Table 1.

**Table 1:** Sociodemographic characteristics of participants (n = 79). NGO: nongovernmental organization

Variables	Frequency	Percentage
Age group (years)		
60‑70	26	32.9%
71‑80	28	35.4%
>80	25	31.6%
Gender		
Male	25	31.6%
Female	54	68.4%
Religion		
Hindu	60	75.9%
Muslim	10	12.7%
Buddhist	5	6.3%
Christian	4	5.1%
Marital status		
Married	7	8.9%
Widow	52	65.8%
Widower	20	25.3%
Family type		
Nuclear	19	24.1%
Joint	60	75.9%
Socioeconomic class (Modified B.G. Prasad Scale)		
Upper	6	7.6%
Upper middle	12	15.2%
Middle	14	17.7%
Lower middle	27	34.2%
Lower	20	25.3%
Comorbidity		
Single	41	51.9%
Multiple	38	48.1%
Type of old-age home		
Government	32	40.5%
NGO	47	59.5%

QOL

**Figure 1:**
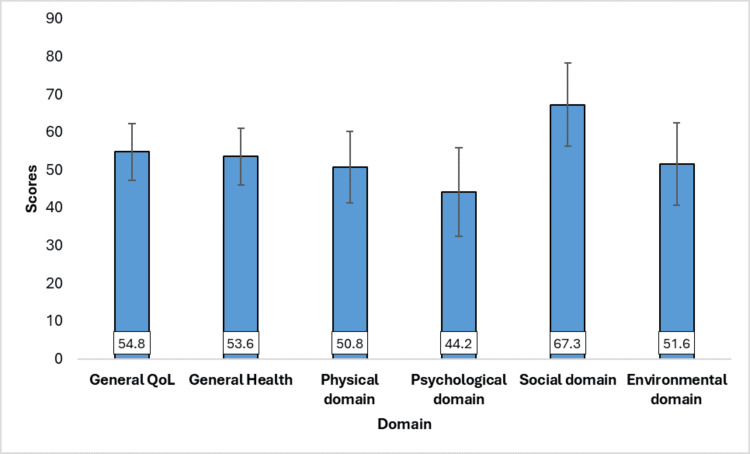
Mean QOL domain scores among the participants. Error bars represent the standard error. QOL: quality of life

The mean general QOL score was 54.8 ± 7.5, and the mean general health score was 53.6 ± 7.5. Among the WHOQOL-BREF domains, the physical health domain had a mean score of 50.8 ± 9.5, whereas the psychological domain had the lowest mean score (44.2 ± 11.7). The social relationships domain recorded the highest mean score (67.3 ± 11.0), followed by the environment domain (51.6 ± 10.9) (Figure 1).

Table 2 presents the comparison of domain scores across participants’ sociodemographic and institutional characteristics. Participants aged ≤70 years had significantly higher physical health domain scores than those aged >70 years (53.5 ± 9.3 vs 48.1 ± 9.6; p = 0.020). Psychological domain scores differed significantly across socioeconomic classes, with participants in the upper/upper-middle socioeconomic class reporting the highest scores (49.3 ± 10.5), followed by those in the middle (42.6 ± 12.6) and lower/lower-middle socioeconomic classes (40.8 ± 12.1) (p = 0.040). No statistically significant differences were observed in the physical health, psychological, social relationships, or environment domain scores according to gender, religion, marital status, family type, or type of old-age home (all p > 0.05). Likewise, the psychological, social relationships, and environment domain scores did not differ significantly between the two age groups, whereas the physical health, social relationships, and environment domain scores did not differ significantly across socioeconomic classes (all p > 0.05).

**Table 2:** Association of sociodemographic variables with QOL domains (n = 79). * Statistically significant NGO: nongovernmental organization; QOL: quality of life

Variable	Physical health	Psychological	Social relationships	Environment
Age group (years)				
≤70 (n = 26)	53.5 ± 9.3	45.4 ± 11.6	66.1 ± 10.4	50.4 ± 9.3
>70 (n = 53)	48.1 ± 9.6	43.0 ± 11.8	68.4 ± 11.5	52.8 ± 12.5
p-value	0.020*	0.395	0.391	0.388
Gender				
Male (n = 25)	49.0 ± 8.2	46.3 ± 11.2	66.6 ± 11.7	51.4 ± 11.2
Female (n = 54)	51.6 ± 10.0	43.2 ± 12.3	67.9 ± 10.2	51.7 ± 10.5
p-value	0.260	0.303	0.616	0.905
Religion				
Hindu (n = 60)	52.4 ± 8.6	46.6 ± 10.7	68.4 ± 13.2	53.3 ± 11.7
Muslim (n = 10)	49.1 ± 9.4	42.9 ± 13.5	65.9 ± 12.5	48.8 ± 10.9
Others (n = 9)	50.9 ± 10.5	43.0 ± 10.9	67.6 ± 7.4	52.7 ± 10.1
p-value	0.530	0.461	0.842	0.519
Marital status				
Married (n = 7)	48.9 ± 10.2	43.4 ± 11.8	68.2 ± 9.5	50.9 ± 10.7
Widowed/widower (n = 72)	52.7 ± 8.7	45.0 ± 11.5	66.4 ± 13.8	52.3 ± 11.1
p-value	0.280	0.726	0.737	0.750
Family type				
Nuclear (n = 19)	49.9 ± 9.8	45.8 ± 11.4	65.4 ± 11.8	53.2 ± 11.7
Joint (n = 60)	51.7 ± 9.1	42.6 ± 11.9	69.2 ± 10.2	50.0 ± 10.1
p-value	0.462	0.305	0.177	0.250
Socioeconomic class				
Upper/Upper middle (n = 18)	52.7 ± 9.1	49.3 ± 10.5	70.2 ± 9.5	51.8 ± 9.2
Middle (n = 14)	49.3 ± 8.5	42.6 ± 12.6	66.5 ± 10.4	53.6 ± 11.3
Lower/Lower middle (n = 47)	50.3 ± 10.9	40.8 ± 12.1	65.1 ± 13.1	49.5 ± 12.3
p-value	0.597	0.040*	0.311	0.461
Type of old-age home				
Government-run (n = 32)	49.4 ± 8.0	42.9 ± 12.6	67.1 ± 11.4	53.6 ± 11.1
NGO-managed (n = 47)	51.8 ± 10.5	45.1 ± 11.1	65.5 ± 10.9	50.3 ± 10.7
p-value	0.288	0.412	0.912	0.186

Table 3 shows the results of multiple linear regression analyses examining the associations between sociodemographic and clinical characteristics and overall QOL and its domains. Six separate regression models were fitted for general QOL, general health, and the physical health, psychological, social relationships, and environment domains. The overall models were not statistically significant for general QOL (R² = 0.056, F = 0.403, p = 0.941), general health (R² = 0.106, F = 0.810, p = 0.620), the physical health domain (R² = 0.138, F = 1.090, p = 0.383), the psychological domain (R² = 0.172, F = 1.410, p = 0.195), the social relationships domain (R² = 0.112, F = 0.861, p = 0.573), and the environment domain (R² = 0.080, F = 0.588, p = 0.819), indicating that the included predictors explained between 5.6% and 17.2% of the variance in the respective outcomes. Among the predictors examined, only two variables demonstrated statistically significant associations with quality-of-life domains. Participants in the upper/upper-middle socioeconomic class had significantly higher psychological domain scores than those in the lower/lower-middle socioeconomic class (B = 11.63, 95% CI: 2.78-20.48, p = 0.011). In addition, participants from joint families had significantly higher social relationships domain scores than those living in nuclear families (B = 8.79, 95% CI: 0.54-17.03, p = 0.037). No significant associations were observed between age, gender, religion, marital status, comorbidity status, and any of the quality-of-life outcomes (all p > 0.05). Overall, although the regression models did not achieve statistical significance, higher socioeconomic status was independently associated with better psychological QOL, whereas living in a joint family was independently associated with better social QOL.

**Table 3:** Multiple linear regression analyses of factors associated with QOL (N = 79). * Statistically significant NGO: nongovernmental organization; QOL: quality of life

Predictor	General QOL		General health		Physical		Psychological		Social		Environmental	
	B (95% CI)	p-value	B (95% CI)	p-value	B (95% CI)	p-value	B (95% CI)	p-value	B (95% CI)	p-value	B (95% CI)	p-value
Age (years)	0.05 (-0.22, 0.32)	0.701	-0.09 (-0.35, 0.17)	0.489	0.26 (-0.07, 0.59)	0.117	-0.04 (-0.43, 0.35)	0.833	0.28 (-0.11, 0.66)	0.154	0.07 (-0.32, 0.46)	0.715
Gender												
Male vs female	1.01 (-3.60, 5.61)	0.664	3.05 (-1.42, 7.52)	0.178	-3.20 (-8.78, 2.38)	0.257	3.13 (-3.55, 9.81)	0.352	-2.86 (-9.40, 3.68)	0.386	-3.32 (-9.91, 3.26)	0.317
Religion												
Muslim vs Hindu	3.34 (-2.95, 9.63)	0.293	1.38 (-4.72, 7.49)	0.652	2.62 (-5.00, 10.25)	0.495	-0.51 (-9.64, 8.61)	0.911	-2.93 (-11.86, 6.01)	0.516	1.09 (-7.90, 10.09)	0.809
Others vs Hindu	-0.83 (-8.12, 6.46)	0.820	-4.56 (-11.64, 2.52)	0.203	4.31 (-4.52, 13.14)	0.334	-1.04 (-11.61, 9.54)	0.845	4.53 (-5.82, 14.88)	0.386	-3.49 (-13.92, 6.93)	0.506
Marital status												
Widow/widower vs married	2.40 (-5.55, 10.35)	0.549	0.26 (-7.46, 7.98)	0.947	-1.40 (-11.03, 8.24)	0.773	8.12 (-3.42, 19.65)	0.165	0.85 (-10.45, 12.14)	0.882	0.43 (-10.93, 11.80)	0.940
Family type												
Joint vs nuclear	2.17 (-3.63, 7.98)	0.457	-0.78 (-6.42, 4.86)	0.784	5.66 (-1.38, 12.69)	0.113	5.40 (-3.02, 13.82)	0.205	8.79 (0.54, 17.03)	0.037*	4.73 (-3.57, 13.03)	0.259
Socioeconomic class												
Middle vs lower/lower-middle	-0.08 (-5.92, 5.77)	0.979	2.26 (-3.42, 7.94)	0.430	-5.42 (-12.51, 1.66)	0.131	-1.48 (-9.95, 7.00)	0.730	-5.69 (-13.99, 2.61)	0.176	1.23 (-7.12, 9.59)	0.769
Upper/upper-middle vs lower/lower-middle	1.07 (-5.03, 7.18)	0.726	2.30 (-3.62, 8.23)	0.441	2.10 (-5.30, 9.49)	0.574	11.63 (2.78, 20.48)	0.011*	2.47 (-6.19, 11.14)	0.571	3.08 (-5.64, 11.80)	0.483
Comorbidity												
Multiple vs single	-0.48 (-4.78, 3.82)	0.825	1.65 (-2.53, 5.82)	0.434	0.35 (-4.87, 5.56)	0.895	-3.78 (-10.02, 2.46)	0.231	-0.16 (-6.27, 5.95)	0.959	1.17 (-4.97, 7.32)	0.704
Type of old-age home												
NGO vs government	0.77 (-3.50, 5.05)	0.719	-3.11 (-7.27, 1.04)	0.139	3.75 (-1.43, 8.93)	0.153	0.90 (-5.30, 7.10)	0.773	2.49 (-3.58, 8.57)	0.415	-3.56 (-9.67, 2.56)	0.250

## Discussion

The present study assessed the QOL of elderly residents of old-age homes in Agartala, Tripura, using the WHOQOL-BREF instrument. Overall, the participants demonstrated moderate QOL across all domains, with the highest mean score observed in the social relationship domain and the lowest in the psychological domain. Younger elderly participants had significantly better physical health, while higher socioeconomic status independently predicted better psychological well-being, and participants who previously belonged to joint families demonstrated better social QOL.

The mean age of the participants was 75 years, and nearly two-thirds were women. This demographic pattern is comparable to studies from Gujarat by Patel and Singh and Maharashtra by Amonkar et al., in which females constituted the majority of institutionalized elderly residents [6,7]. The predominance of women in old-age homes may be attributed to their longer life expectancy, higher rates of widowhood, greater financial dependency, and reduced family support following the death of their spouse. In many parts of India, elderly women remain particularly vulnerable to social isolation because they are less likely to possess independent financial resources or property, making institutional care a more feasible option than family-based care.

The present study found that the social relationship domain recorded the highest QOL score despite all participants residing in institutional care. This finding appears counterintuitive because institutionalization is often considered a risk factor for social isolation. However, it may reflect the supportive social environment within old-age homes, where residents interact daily with peers who share similar life experiences, participate in communal meals and recreational activities, and receive regular assistance from caregivers. Such structured social interactions may alleviate loneliness associated with living alone and foster companionship and a sense of belonging. Comparable results have been documented in the literature, indicating that social relationships are among the higher-performing domains of the WHOQOL-BREF [17]. Furthermore, a recent systematic review concluded that opportunities for meaningful social interaction, participation in recreational activities, and supportive relationships with caregivers are major determinants of QOL among nursing home residents [5]. However, a high social relationship score should not be interpreted as evidence of complete psychosocial well-being. The WHOQOL-BREF social domain primarily assesses satisfaction with personal relationships, social support, and social contacts but does not capture deeper emotional constructs such as grief, purpose in life, autonomy, or existential distress. Consequently, residents may report satisfactory social interactions while simultaneously experiencing emotional distress.

In contrast, the psychological domain recorded the lowest mean score, highlighting a striking discrepancy between social connectedness and emotional well-being. This paradox has been increasingly recognized in geriatric research, where institutionalized older adults often maintain adequate social contact but continue to experience depression, anxiety, loneliness, feelings of abandonment, and loss of personal identity [9,18]. In Tripura, the traditional emphasis on family unity and multigenerational living has been crucial to caring for older adults. Moving to an old-age home can therefore be an emotionally challenging transition. While individuals may socially adjust to life in the facility, the ongoing impact of being away from family, experiencing loss, diminishing independence, and having less control over their lives can still negatively influence their mental health. Previous studies have shown that emotional support from family members contributes differently to mental health than social interactions with peers, explaining why satisfactory social relationships may coexist with poor psychological outcomes [18,19]. These findings suggest that improving social activities alone may not address residents’ psychological needs. Therefore, mental health screening, counseling, cognitive stimulation, and occupational therapy should complement social care to improve QOL.

Participants aged ≤70 years had significantly higher physical domain scores than those aged >70 years. This finding was expected because advancing age is associated with progressive declines in muscle strength, mobility, balance, and functional independence, together with increasing multimorbidity and frailty. Comparable associations between increasing age and poorer physical QOL have been reported in recent community-based studies from Bihar and South India [15,20]. Although age lost significance in the multivariable analysis, the observed trend supports the well-established influence of aging on physical functioning and emphasizes the need for interventions promoting healthy aging, regular physical activity, and rehabilitation.

Similarly, participants from higher socioeconomic classes exhibited significantly better psychological QOL in both univariate and multivariable analyses. This association likely reflects the cumulative influence of lifelong socioeconomic advantages rather than current living conditions. Individuals with higher socioeconomic status generally have better educational attainment, financial security, health literacy, and access to health care throughout their lives, all of which contribute to greater resilience and more effective coping with age-related stressors [21]. Conversely, residents from lower socioeconomic backgrounds may experience greater financial insecurity, reduced autonomy, and concerns regarding health care expenses or dependence on institutional support, thereby increasing their psychological distress. In Tripura, where a substantial proportion of older adults depend on limited pensions and family support, socioeconomic disadvantage may further compound feelings of uncertainty and loss of control after institutionalization. Similar associations have been reported in community-based Indian studies and the international literature, where socioeconomic disadvantage consistently predicts poorer mental health and reduced QOL among older adults [5,12,15]. These findings emphasize that improving mental well-being among elderly residents requires not only institutional support but also policies that address lifelong social and economic inequalities.

The findings also demonstrated that participants who belonged to joint families before institutionalization had significantly better social quality-of-life scores after adjusting for other variables. Individuals raised in larger family systems may retain stronger interpersonal skills, broader social networks, and more frequent contact with relatives even after entering residential care. Previous Indian studies have similarly shown that elderly individuals with stronger family support generally report better social and emotional well-being than those from nuclear families [12,13]. This finding emphasizes the continuing influence of lifelong family relationships on successful aging. No significant differences were observed according to gender, religion, marital status, comorbidity status, or type of old-age home. Similar findings have been reported in an institutional study from Kerala, suggesting that residents experience relatively comparable living conditions irrespective of these characteristics [17]. However, the modest sample size may have limited our ability to detect smaller differences between subgroups.

This study had several limitations that should be considered while interpreting the findings. Although randomly selected old-age homes and a validated WHOQOL-BREF instrument were used, the cross-sectional design precludes causal inference, and the relatively small sample size from a single city limits the generalizability of the findings. Furthermore, several important determinants of QOL among older adults, including functional status, cognitive impairment, depression, duration of institutionalization, medication burden, sleep quality, and physical activity, were not assessed. The omission of these variables may explain the modest explanatory power of the regression models and could have resulted in residual confounding. Additionally, the exclusion of critically ill individuals and reliance on self-reported responses may have introduced selection and response biases. Future multicenter longitudinal studies incorporating these clinical, functional, and psychosocial factors are warranted to provide a more comprehensive understanding of the QOL of institutionalized older adults.

## Conclusions

The present study found that elderly residents of old-age homes in Agartala had relatively higher QOL scores in the social relationship domain, whereas the psychological domain recorded the lowest scores among the WHOQOL-BREF domains. Younger age was associated with better physical QOL, while higher socioeconomic status and previous joint family living were independently associated with better psychological and social QOL, respectively. These findings provide region-specific evidence on the QOL of institutionalized older adults in Tripura and may assist in planning comprehensive geriatric care and supportive services for this population. Further longitudinal and qualitative studies are needed to explore additional factors influencing QOL in this population.

## Appendices

Appendix A: Study questionnaire 

**Table 4:** Questionnaire used for data collection WHOQOL-BREF: World Health Organization Quality of Life-BREF

Quality of life of the elderly residing in old-age homes, Tripura						
Participant ID						
Date of interview						
Name of old-age home						
Section I						
Age (completed years)		.............years				
Gender		Male/Female/Other				
Religion		Hindu/Muslim/Christian/Buddhist/Other				
Marital status		Married/Widow/Widower/Divorced or separated/Never married				
Residence before admission		Urban/Rural				
Family type before admission		Nuclear/Joint/Extended				
Monthly income (₹)						
Socioeconomic class (Modified BG Prasad)		Upper/Upper Middle/Middle/Lower Middle/Lower				
Any chronic disease diagnosed		Yes/No				
		If yes, specify the disease.				
Section II (WHOQOL-BREF)						
A. General quality of life						
Question no.	Question	Very poor (1)	Poor (2)	Neither poor nor good (3)	Good (4)	Very good (5)
1	How would you rate your quality of life?					
Question No.	Question	Very dissatisfied (1)	Dissatisfied (2)	Neither satisfied nor dissatisfied (3)	Satisfied (4)	Very satisfied (5)
2	How satisfied are you with your health?					
B. Experienced during the last two weeks						
Question no.	Question	Not at all (1)	A little (2)	A moderate amount (3)	Very much (4)	An extreme amount (5)
3	To what extent do you feel that physical pain prevents you from doing what you need to do?					
4	How much medical treatment do you need to function in your daily life?					
5	How much do you enjoy life?					
6	To what extent do you feel your life to be meaningful?					
7	How well are you able to concentrate?					
8	How safe do you feel in your daily life?					
9	How healthy is your physical environment?					
C. Ability to do certain things						
Question no.	Question	Not at all (1)	A little (2)	Moderately (3)	Mostly (4)	Completely (5)
10	Do you have enough energy for everyday life?					
11	Are you able to accept your bodily appearance?					
12	Do you have enough money to meet your needs?					
13	How available to you is the information that you need in your day-to-day life?					
14	To what extent do you have the opportunity for leisure activities?					
Question no.	Question	Very poor (1)	Poor (2)	Neither poor nor good (3)	Good (4)	Very good (5)
15	How well are you able to get around?					
D. Satisfaction with life aspects						
Question no.	Question	Very dissatisfied (1)	Dissatisfied (2)	Neither satisfied nor dissatisfied (3)	Satisfied (4)	Very satisfied (5)
16	How satisfied are you with your sleep?					
17	How satisfied are you with your ability to perform your daily living activities?					
18	How satisfied are you with your capacity for work?					
19	How satisfied are you with yourself?					
20	How satisfied are you with your personal relationships?					
21	How satisfied are you with your sex life?					
22	How satisfied are you with the support you get from your friends?					
23	How satisfied are you with the conditions of your living place?					
24	How satisfied are you with your access to health services?					
25	How satisfied are you with your transport?					
E. Frequency of negative feelings						
Question no.	Question	Never (1)	Seldom (2)	Quite often (3)	Very often (4)	Always (5)
26	How often do you have negative feelings such as a blue mood, despair, anxiety, or depression?					
